# This Bioengineer Transforms Food Waste into Haute Cuisine

**DOI:** 10.1021/acscentsci.6c00297

**Published:** 2026-02-25

**Authors:** Kristel Tjandra

## Abstract

Chef-turned-scientist is fighting the food crisis with fungal fermentation.

## Graphical abstract

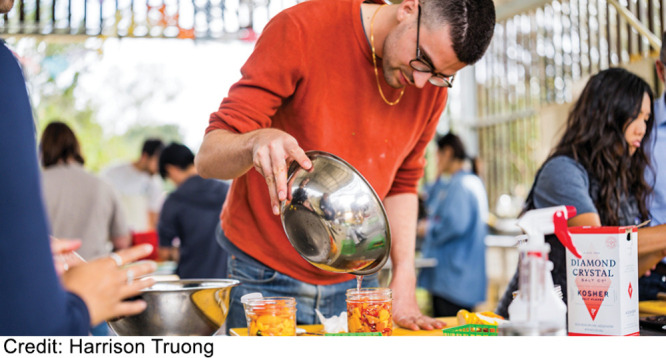

Filamentous fungi, like mushrooms and molds, are essential components in a wide variety of fermented food products. From cheese and chocolate to miso and beer, these organisms not only produce unique flavors but also can transform inedible plant matter into nutritious treats.

**Figure:**
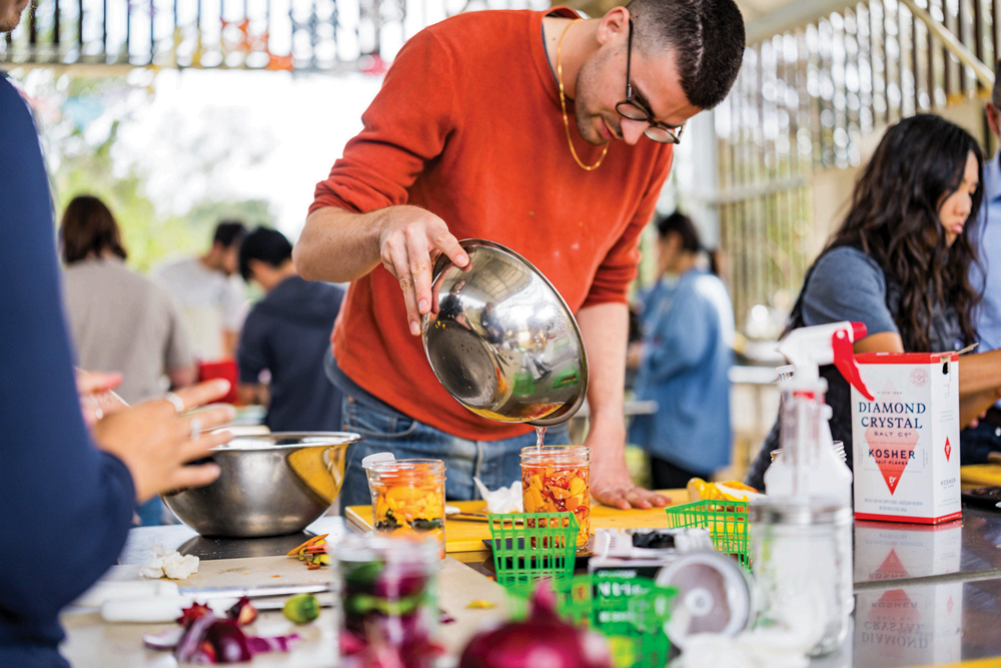
Chef-turned-bioengineer Vayu Hill-Maini uses fungal fermentation to make food waste delicious. Credit: Harrison Truong.

Unlike bacteria or yeasts that mainly turn simple sugars into alcohols or acids, filamentous fungi are adept at breaking down complex substrates. This makes the fungi suitable for converting biowaste—such as fruit peels, cereal straws, and pulp—into food.

While fermented products have been a part of our diet for millennia, the genetics of fungi that turn biowaste into food have not been studied in detail, let alone engineered for gastronomy. This untapped potential inspired Vayu Hill-Maini, a chef-turned-bioengineer at Stanford University, to focus his research on developing new biological tools that harness fungi’s ability to turn byproducts of food production into food. To combine advancements in biotechnology and culinary creativity, Hill-Maini partnered with Ramón Perisé—a two-Michelin-star chef—to explore how fungal fermentation can inspire new and sustainable menus.

Kristel Tjandra talked to Hill-Maini about this unique collaboration. This interview was edited for length and clarity.

## How did you get into fermentation?

I was a chef before I became a scientist. And it was through my experiences in the kitchen—smelling, learning, experimenting, and cooking—that I became fascinated by science and engineering as a way to look at food through a new lens. I learned that a piece of matter could be engineered, modified, and improved.

I choose to focus on fermentation because it is one of the most powerful ways to create new flavors and nutrition, used by people for thousands of years. It is the original biotechnology, where people use microbes to create products that have cultural, economic, and planetary value.

## What’s so special about fermentation with fungi?

When we think about fermentation, most people think about taking crops we eat, such as wheat or grapes, and transforming or enhancing them to create new products, such as beer and wine. What is unique about fungi is that they can take things that are difficult to digest, or maybe not even edible to begin with, and completely transform them into human food.

## Such as?

A good example of this is soybeans. Soybeans have a lot of digestive inhibitors—that is, molecules and proteins that can interfere with human digestion—so they’re a little bit difficult to eat in their raw form. Fermentation, however, removes and transforms [those digestive inhibitors]. When soybeans are turned into miso or soy sauce, you get an incredible umami, savory deliciousness.

A more extreme example is oncom, a ferment that’s made traditionally in Indonesia on the island of Java. Oncom is unique in that it’s made from waste ingredients that are coming out of food processing, such as the [peanut] press cake from peanut oil production and the cellulose-rich soybean biomass. Those waste products are fermented through the fungus *Neurospora intermedia* and turned into a meat substitute known as oncom. When we studied the genomic diversity of this fungus, we got a really interesting perspective on how people use fungi to control and direct the flavor of this traditional food.

## How is your lab working on making fungi-fermented food delicious?

In our lab, we ask the question: What could fungal foods 2.0 look like? And to answer this, we are characterizing fungi, understanding how they break down materials and generate flavors and nutrients, and then developing tools to engineer fungi using technologies like CRISPR-Cas9. The final piece of the puzzle is to think about how we actually create foods that people want to eat, bridging flavor and human needs with our understanding of [fungal] genomes.

## Tell me more about bringing a Michelin-starred chef to your lab.

I think chefs are uniquely positioned to lead the cultural and aesthetic part of this challenge. They’re innovators who dedicate their lives to understanding food as material, as culture, and a tool for storytelling. And instead of having scientists develop something in the lab and coming to a chef and saying, “Hey, look what we made. Can you put this on your menu?” We want to integrate chefs and scientists from the very beginning.

I first met Ramón Perisé, who is the head of innovation and development at Mugaritz, when I was a sophomore in college. Mugaritz is probably one of the most innovative restaurants in the world over the last 20 years. I realized from talking to him that what they were doing at Mugaritz was exactly what I wanted to do: bringing together scientists, chefs, and artists to innovate. So I invited him.

**Figure:**
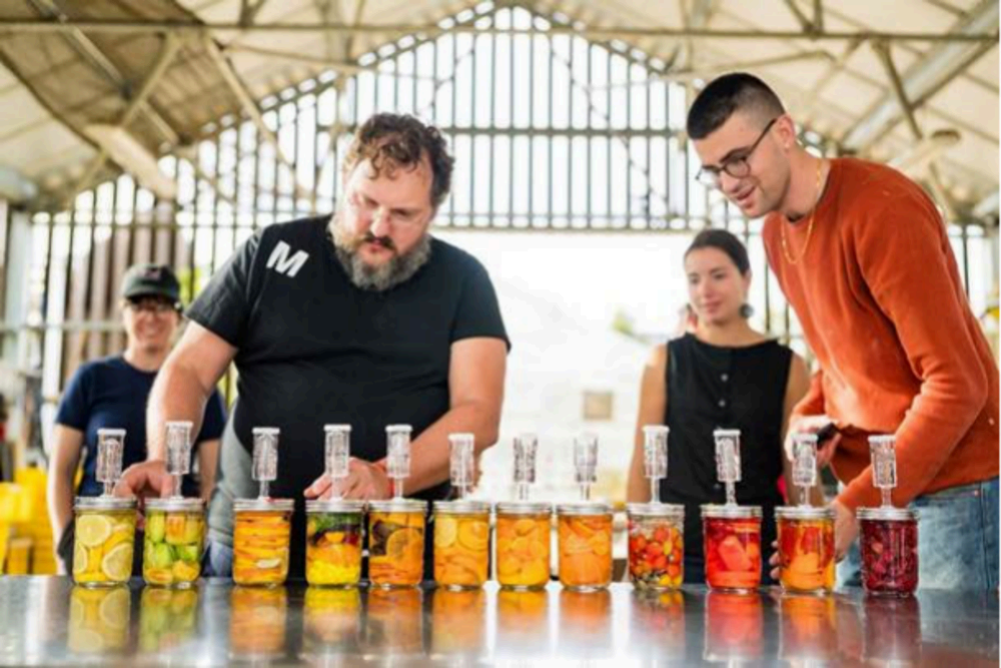
Ramón Perisé (left) of the restaurant Mugaritz and Vayu Hill-Maini lead a fermentation workshop at Stanford as part of the Chef-in-Residence program, which brings scientists and chefs together. They collected seasonal fruits and vegetables grown at the Stanford farm and fermented them to learn about preserving the fall’s harvest for the future. Credit: Harrison Truong.

## What have you been able to do together through your lab’s Chef-in-Residence program?

Ramon was already in our lab for 3 weeks in the fall of 2025, and he’ll return for 3 weeks in spring 2026. He’s given a public lecture to share his vision for gastronomy and what they do at Mugaritz. He’s also been engaging directly with students, teaching workshops on campus on fungi and fermentation.

We’ve also been using fungi in the kitchen next to our lab to make food. We are thinking about the connection between genetics and flavor, and how we can link those two together. I’ve been showing Chef Ramón our CRISPR experiments, where we are developing very early prototypes of modified fungi.

## What kind of modifications are we talking about?

We are the first to develop genetic editing methods to understand the intriguing metabolism of the edible fungus *Neurospora intermedia—*how it builds flavor and texture—and also engineer it to enhance its properties, focusing on aesthetics and nutrition. One of the key things we have already done is to change its color. While the standard fungus is orange, we have generated mutants that are white, which are suitable for different applications where we don’t want the food to be orange.

I think one of the challenges here in Silicon Valley with food innovation is that people have made really cool technology, but this stuff tastes like shit and nobody wants to eat it. I think it’s really important that we take into account the perspectives of chefs. Let’s think about deliciousness and flavor at the outset, not as an afterthought.

We are also working with edible mushrooms, such as oyster mushrooms, in our lab. We have shown Ramon our initial successful experiments where we have expressed fluorescent proteins in the edible fruiting body of a model mushroom, which now enables us to focus on flavor as a next step. The Mugaritz team was blown away by these findings!

## What foods have you made together?

We have made a few different things. One of the really exciting ones is a plant-based beverage that only has two ingredients: grains and fungus. During the growth, fungal enzymes hydrolyze the starch in the grains into sugars that give a delicious sweetness. They also change the texture by liquefying the starch and, finally, make fruity aromas reminiscent of bee pollen and flowers and ripe fruit. We have made delicious sausages; burgers; and a plant-based cheese that [can be grated], like pecorino, from fungal fermentation of waste products. And Ramón and others from Mugaritz have tasted these and given some very useful feedback for us on how to improve the flavor, texture, and presentation.

## Was there anything that you tried that didn’t quite work?

We have made some things that did not work—for example, growing a particular strain of fungus on just rice. We initially expected to get a sweet product, but instead we got a bitter and weird, off-flavor product. We quickly learned that it is not a viable path. Moving forward, we will incorporate genetics in these experiments to try to knock out certain flavors and aromas, really to connect our molecular understanding with the sensory profiles. In this process, we are learning how to build a shared language between the lab and the kitchen.

## What are you hoping to achieve through this collaboration and through your work on fungal fermentation in general?

We’re facing a crisis in our food system. The food system contributes to about 30% of all greenhouse gas emissions, and we have to fundamentally reimagine what we eat and how we produce it.

Fungi have been a staple of the human diet since neolithic times. We are deeply familiar with them through their uses as mushrooms as well as in fermented foods such as cheese, soy sauce, miso, tempeh, and more.

But fungi are also a really promising alternative to animal meat with their low environmental impact, high nutrition, low land use, and massive greenhouse gas reductions. Fungi have a meat-like texture encoded in their biology; molds and mushrooms grow as filaments, which we perceive as meat-like in our mouth. In some cultures, the word for fungus is related to that of animal or meat.

So, if we really want to onboard fungi as a future food source, we have to think about how we can maximize their potential, and even engineer and program them for the outputs we want. Without flavor, deliciousness, and nutrition at the center, none of the green sustainability stuff will matter.

*Kristel Tjandra is a freelance contributor to* Chemical & Engineering News, *an independent news publication of the American Chemical Society.*

